# Screening of reference genes for microRNA analysis in the study of solider caste differentiation of Formosan subterranean termite *Coptotermes formosanus* Shiraki

**DOI:** 10.1038/s41598-023-35926-7

**Published:** 2023-06-09

**Authors:** He Du, Runmei Huang, Dasong Chen, Chaofu Huang, Huan Zhang, Zhiqiang Lia

**Affiliations:** 1 Guangdong Key Laboratory of Integrated Pest Management in Agriculture, Guangdong Public Laboratory of Wild Animal Conservation and Utilization, Institute of Zoology, Guangdong Academy of Sciences, Guangzhou, 510260 China; 2 Nanning Institute of Termite Control, Nanning, 530023 China; 3 State Key Laboratory of Integrated Management of Pest Insects and Rodents, Institute of Zoology, Chinese Academy of Sciences, Beijing, 100101 China

**Keywords:** Biological techniques, Genetics, Molecular biology, Zoology

## Abstract

The soldier caste differentiation is a complex process that is governed by the transcriptional regulation and post-transcriptional regulation. microRNAs (miRNAs) are noncoding RNAs that control a wide range of activities. However, their roles in solider caste differentiation are barely studied. RT-qPCR is a powerful tool to study the function of genes. A reference gene is required for normalization for the the relative quantification method. However, no reference gene is available for miRNA quantification in the study of solider caste differentiation of *Coptotermes formosanus* Shiraki. In this research, in order to screen the suitable reference genes for the study of the roles of miRNAs in solider caste differentiation, the expression levels of 8 candidate miRNA genes were quantified in the head and thorax + abdomen during soldier differentiation. The qPCR data were analyzed using geNorm, NormFinder, BestKeeper, ΔC_t_ method and RefFinder. The normalization effect of the reference genes was evaluated using the *let-7-3p*. Our study showed that *novel-m0649-3p* was the most stable reference gene, while *U6* was the least stable reference gene. Our study has selected the most stable reference gene, and has paved the way for functional analysis of miRNAs in solider caste differentiation.

## Introduction

Termites are eusocial insects with division of labor as one of their distinct characteristics. This feature of dividing labor among colony members allows termites to form a rather complex social system, and enables them to improve the efficiency of operating the termite colony, thus facilitating termites’ evolutionary and ecological success^1^. The termite society typically consists of different castes, such as reproductives, workers and soldiers. In particular, the soldier caste is the defensive caste inside the society^2^. Normally, the colony could maintain a relative stable ratio of soldier caste^3^, but when the soldier ratio is below the regular ratio for the species, new soldier could form^4^. The formation of soldier is induced by high juvenile hormone (JH) titer in the hemolymph^5–7^. Juvenile hormone analog (JHA) could also artificially induce formation of soldiers. JH drives the differential expression of the genes, and triggers a complex of gene cascade during the soldier morphogenesis. For example, hexamerin is synthesized massively during the process and identified as the key regulation protein to buffer the function of JH^8^. In addition, the classical signaling pathways, such as JH signaling pathway, TGFβ signaling pathway, and insulin signaling pathway, are involved in soldier formation regulation^9–11^. However, the mechanism of soldier caste differentiation is mainly focused at the transcriptional level, the roles of microRNAs (miRNAs) are barely studied. 

miRNAs are noncoding RNAs with the length of ~ 21–24 nt. They play important roles in RNA silencing and the post-transcriptional regulation through repression of gene translation or degradation of mRNA transcripts^12^. miRNAs regulate behavior, development, reproduction and immunity in insects^13–15^. In particular, miRNAs mediate the social immunity of termites to pathogens^16^. The caste differentiation of termites involves a dynamic change of gene cascade. At a certain stage of the transformation, the high expressed genes need to be cleaned up or maintained at relative low expression. Considering the significance of miRNAs in a wide range of physiological activities, they may serve functions in soldier caste differentiation. Up to date, only two pieces of research on the roles of miRNAs in caste differentiation in termites were completed. Itakura et al.^17^ sequenced the miRNAs in worker caste of *Coptotermes formosanus* Shiraki and *Reticulitermes speratus* (Kolbe). Matsunami et al.^18^ reported the differential expression of miRNAs in the workers and soldiers of *R. speratus*, indicating the possible involvement of miRNAs in caste differentiation in termites. However, the roles of miRNAs in soldier differentiation are not quite clear, and have to be confirmed with gene expression analysis.

RT-qPCR is a standard technique to quantify gene expression level, either using the absolute quantification or the relative quantification method. The latter needs a reference gene to normalize the qPCR data. Unproper choice of reference gene has a negative effect on data analysis, such as increasing the error or even not detecting of gene expression pattern^19–21^. Although the housekeeping genes, such as 18S, β-actin, 5S rRNA and U6 snRNA, have been widely used for qPCR data normalization, their expression levels vary considerably in certain experiment conditions^22–25^. Since choice of reference genes depends on the conditions of the experiment, there are no universal reference genes for all the qPCR data analysis. The ideal way to choose reference gene is to screen the reference genes for the specific experiment. Hence, many studies have been conducted to screen the suitable reference genes for different species and under various experimental conditions ^26–34^. However, there is no reference gene available for miRNA quantification in the study of solider caste differentiation of *C. formosanus*.

We have sequenced the miRNAs of *C. formosanus*, and through checking the miRNome data, we identified 8 candidate reference genes which had the middle expression level and were least influenced by the stimulation of a JHA, methoprene. In order to test the suitability of the reference genes, the expression profile of a conserved miRNA, *let-7*, was quantified. The objective of this project is to screen the suitable reference gene for miRNA quantification in the study of the solider caste differentiation.

## Materials and methods

### Bioassay and sample preparation

Termites were collected from Shangchong Fruit Park, South China University of Technology, Guangdong Tree Park, and Dafushan Forest Park, Guangzhou, China. Termites were kept in the laboratory at room temperature and used within two weeks.

In order to induce solider formation, workers were treated with methoprene^35^. Acetone solution of methoprene was dripped evenly onto filter paper to make the concentration of methoprene as 1000 ppm. Two pieces of filter paper (32 mm in diameter) were placed in a Petri dish (35 mm in diameter). The paired filter paper was then wet by adding 240μL distilled water. Twenty workers were added to each Petri dish. The Petri dishes were placed in a sealed container. A piece of wet paper towel was placed in the container to maintain the moisture level inside. The experimental set was placed in an incubator at 28 °C. Four colonies were used.

Termites were collected after feeding on methoprene for the following days: 1st day, 5th day, 10th day, and 15th day. Twenty workers were also collected just before the methoprene bioassay (which was designated as 0D). So, there were five samples for each colony (0D, 1D, 5D, 10D, 15D). The heads of the termites were cut with a scalpel. The head and thorax + adbodan were put in 100 μL of QIAzol Lysis Reagent (Qiagen, Hilden, Germany) separately. The tubes containing the termite were immersed into liquid nitrogen immediately after collection, and then stored at −80 °C. The head is the place where the JH is synthesized. The thorax + abdomen is where the fat body is located, as the fat body responses most obviously after JHA treatment^44^. So, we split the termites into two different body parts, and screened the reference genes separately.

### miRNA extraction and quantification

miRNA was extracted using the RNeasy Mini Kit (Qiagen, Hilden, Germany) following the manufacturer’s protocol. The concentration of the RNA was measured with the TGem Spectrophotometer Plus (Tiangen, Beijing, China). The integrity of the RNA was confirmed using agarose gel electrophoresis. RNA was reverse-transcribed with stem-loop RT primers using M-MuLV Reverse Transcriptase (NEB, Ipswich, Massachusetts, United States). The stem-loop RT primers were designed according to Chen et al.^36^ (Table 1). The 10 μL transcription system contained the following ingredients: 2.5 μL M-MuLV Reverse Transcriptase (200 U/uL), 1 μL M-MuLV buffer, 0.7 mM dNTP, 30 nM stem-loop RT primer, 2 U RNasin® Ribonuclease Inhibitor (Promega, Madison, Wisconsin, United States), and 1 μg RNA. Probe-based method was used to conduct the qPCR. The probes were synthesized with 5’-FAM and 3’-MGB modifications (TsingKe, Beijing, China) (Table 1). qPCR was conducted using Luna® Universal Probe qPCR Master Mix (NEB, Ipswich, Massachusetts, United States). The 10 μL qPCR system was as follows: 5 μL Luna universal probe qPCR master mix, 1.5 μM forward primer, 0.7 μM reverse primer, 0.2 μM probe, and 0.7 μL cDNA. The qPCR was conducted on BIO-RAD CFX Connect (Bio-Rad, California, USA). The program for amplification: denaturation at 95℃ for 1 min, with 40 cycles of denaturation at 95℃ 15 s, annealing/extension at 60℃ for 30 s.

**Table 1:** The candidate reference genes, the target gene, the stem loop primers, the probes, the forward primers, and the PCR efficiency.

RNA	RNA sequence	Stem loop primer	Probe	Forward primer	E (%)	R^2^
*miR-193-3p*	UACUGGCCUGCUAAGUCCCAAG	GTCGTATCCAGTGCAGGGTCCGAGGTATTCGCACTGGATACGACCTTGGG	(FAM)ATACGACCTTGGGACT(MGB)	CGTGTACTGGCCTGCTAAGT	113.3	0.997
*miR-971-5p*	CACUCUAAGCUCGAACACCAAGC	GTCGTATCCAGTGCAGGGTCCGAGGTATTCGCACTGGATACGACGCTTGG	(FAM)ATACGACGCTTGGTG(MGB)	GCGTCACTCTAAGCTCGAACA	118.0	0.992
*miR-3049-5p*	UCGGGAAGGCAGUUGCGGCGGACU	GTCGTATCCAGTGCAGGGTCCGAGGTATTCGCACTGGATACGACAGTCCG	(FAM)ATACGACAGTCCGCC(MGB)	TCGGGAAGGCAGTTGCG	100.5	0.991
*miR-216-5p*	AAAUAUCAGCUGGUAAUUCUGA	GTCGTATCCAGTGCAGGGTCCGAGGTATTCGCACTGGATACGACTCAGAA	(FAM)TGGATACGACTCAGAAT(MGB)	CCGAGCGAAATATCAGCTGGTAA	114.5	0.999
*miR-7-3p*	CAAGAAAUCACUCAUCUUCCU	GTCGTATCCAGTGCAGGGTCCGAGGTATTCGCACTGGATACGACAGGAAG	(FAM)TGGATACGACAGGAAG(MGB)	CGACGCCAAGAAATCACTCAT	106.4	0.995
*novel-m0649-3p*	CCUGUAUAUGGUCACUCUCCU	GTCGTATCCAGTGCAGGGTCCGAGGTATTCGCACTGGATACGACAGGAGA	(FAM)TGGATACGACAGGAGAG(MGB)	CGTGGAGCCTGTATATGGTCAC	110.2	0.995
*miR-2788-3p*	CAAUGCCCUUGGAAAUCCCA	GTCGTATCCAGTGCAGGGTCCGAGGTATTCGCACTGGATACGACTGGGAT	(FAM)TGGATACGACTGGGATT(MGB)	CGAGGTCAATGCCCTTGGAA	112.2	0.985
*U6*	ACUAAAAUUGGAACGAUACAGAGAAGAUUAGCAUGGCCCCUGCGCAAGGAUGACACGCAAAAUCGUGAAGCGUUCCACAUUUUU	GTCGTATCCAGTGCAGGGTCCGAGGTATTCGCACTGGATACGACAAAAAT	(FAM)CTGGATACGACAAAAATG(MGB)	GCAAAATCGTGAAGCGTTCCA	104.0	0.995
*let-7-3p*	CUGUACAACUUGCUAACUUUCC	GTCGTATCCAGTGCAGGGTCCGAGGTATTCGCACTGGATACGACGGAAAG	(FAM)TGGATACGACGGAAAGT(MGB)	TGCGACCTGTACAACTTGCTAA	105.6	0.995

Eight genes were chosen as candidate reference genes: *miR-193-3p*, *miR-971-5p*, *miR-3049-5p*, *miR-216-5p*, *miR-7-3p*, *novel-m0649-3p*, *miR-2788-3p*, *U6*. Those genes were chosen from the miRNome data of *C. formosanus*. They were screened because they had middle expression levels and were least influenced by the methoprene treatment according to the analysis of the miRNome data. A conserved miRNA, *let-7-3p*, was chosen as the target gene to evaluate normalization results of the candidate reference genes. A combination of 9 genes × 5 sampling points (0D, 1D, 5D, 10D, 15D) was tested in one qPCR run. So, 4 coloines × 2 body parts = 8 qPCR runs were conducted in total.

### Data analysis

To test the effect of body part and sampling time on C_t_ values, two-way analysis of variance (ANOVA) was used to analyze C_t_ values for each candidate reference gene, with body part and sampling time as two factors. The gene whose C_t_ value is influenced by the bioassay date will be screened out for further analysis, because using this kind of gene as reference gene may skew the normalization results. To test the suitability of the genes as the candidate reference genes, the C_t_ values of the candidate reference genes were analyzed using the four methods: geNorm^37^, NormFinder^38^, BestKeeper^39^, and ΔC_t_ method^40^. The C_t_ value was also analyzed by RefFinder to get a comprehensive stability value^41^. After confirming the suitable genes, correlation was conducted between most stable reference genes and other reference genes to find out the suitable combination to normalize the target gene^19^. To validate the screened reference genes, the expression levels of *let-7-3p* were compared among different sampling dates through normalizing to difference reference genes. The C_t_ value of the target gene was normalized to the C_t_ value of the most stable reference gene, the least stable reference gene and the combination of two reference genes screened out, respectively^37,42,43^. The relative expression levels were log_2_ transformed, and then analyzed with one-way ANOVA.

## Results

### Expression profiles of candidate reference genes

**Figure 1:**
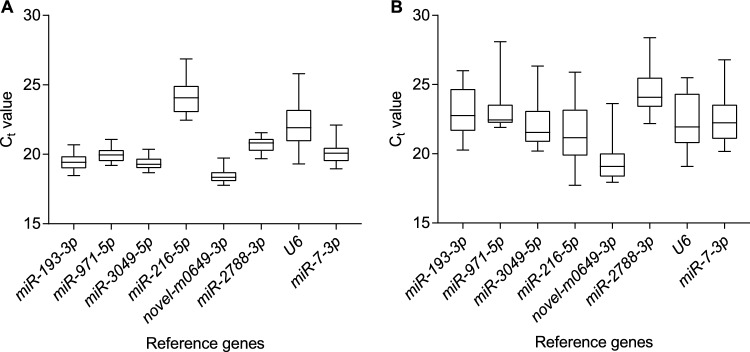
Expression levels of the 8 candidate reference genes in the methoprene bioassay in *Coptotermes formosanus* Shiraki. The median, the upper and lower quartile are shown in the box plots. The expression levels of two body parts are presented separately. (**A**) The epxresssion levels of the 8 candidate reference genes in the head, (**B**) The epxresssion levels of the 8 candidate reference genes in the thorax + abdomen.

The efficiency of the primers is summarized in Table 1. The C_t_ values of the 8 candidate reference genes are shown in Fig. 1. Both the C_t_ values from the head and those from the abdomen + thorax had similar range (Fig. 1). However, more variation was observed in the C_t_ values in the thorax + abdomen compared to that in the head. According to the two-way ANOVA analysis of the C_t_ value for each gene, no effect of sampling time on the C_t_ values was observed for all the genes (Table S1.). Therefore, no genes were excluded for further analysis.

### Expression stability of candidate reference genes in the head

**Figure 2:**
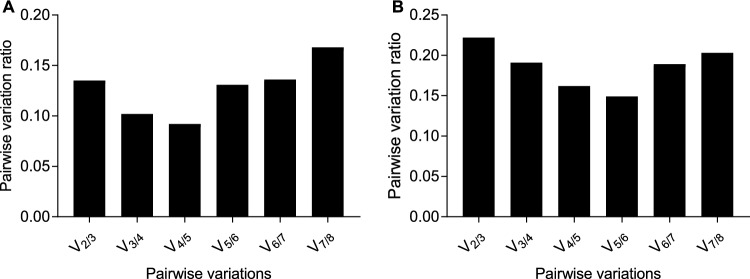
Pairwise variation analysis to determine the optimal number of reference genes using geNorm. (**A**) The pairwise variation analysis result in the head, (**B**) The pairwise analysis result in the thorax + abdomen.

**Figure 3:**
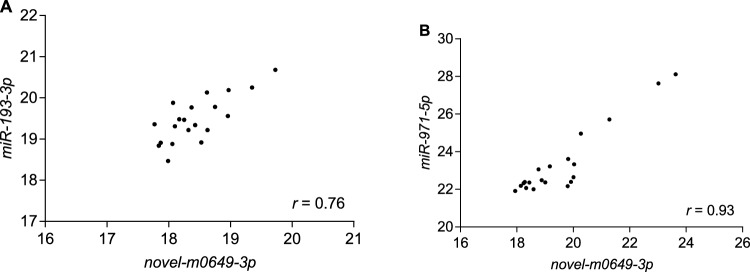
Correlation of the C_t_ values of the two most stable reference genes. (**A**) Correlation of the C_t_ values of the two most stable reference genes in the head. (**B**) Correlation of the C_t_ values of the two most stable reference genes in the thorax + abodman.

Different algorithms returned the results slightly differently (Table 2). According to the comprehensive analysis tool RefFinder, *novel-m0649-3p* was the most stable gene in the head. ΔC_t_ method, NormFinder and geNorm also ranked *novel-m0649-3p* as the most stable gene. However, the result of BestKeeper was slightly different. BestKeeper reports two criteria: SD and [r] as the stability values. *Novel-m0649-3p* ranked as the most stable genes according to SD criterion. However, *novel-m0649-3p* only ranked as the third most stable gene based on the value of [r]. *miR-216-5p* was the most stable gene according to [r]. Although the most stable gene was different, *U6* ranked as the least stable gene according to all of the five algorithims.geNorm calculates the optimal number of genes required for normalization of gene expression. According to pairwise variation analysis, *novel-m0649-3p* and *miR-193-3p* was enough for normalization in the head, as V_2/3_ = 0.135, which was less than the threshold line (0.15) required by the algorithm (Fig. 2A). As suggested by Babion et al*.*^19^, if two genes are highly correlated (*r* > 0.9), i.e. having similar expression pattern, using the combination of two genes to conduct normalization will not result in less technical variation of the target gene than using single gene. The practicability of using the combination of *novel-m0649-3p* and *miR-193-3p* for normalization was also demonstrated by the correlation coefficient between those two genes (*r* = 0.76), which was less than 0.9 (Fig. 3A).

**Table 2:** Ranking of the stability values of the 8 candidate reference genes by the five algorithms in the head.

RefFinder		ΔC_t_		NormFinder		geNorm		BestKeeper				
Gene	GM	Gene	SV	Gene	SV	Gene	SV	Gene	SD	Gene	[r]	*p* value
*novel-m0649-3p*	1.00	*novel-m0649-3p*	0.68	*novel-m0649-3p*	0.178	*novel-m0649-3p*	0.374	*novel-m0649-3p*	0.40	*miR-216-5p*	0.922	0.001
*miR-193-3p*	2.11	*miR-193-3p*	0.74	*miR-193-3p*	0.429	*miR-193-3p*	0.374	*miR-971-5p*	0.41	*U6*	0.918	0.001
*miR-971-5p*	2.91	*miR-971-5p*	0.78	*miR-7-3p*	0.443	*miR-971-5p*	0.420	*miR-3049-5p*	0.42	*novel-m0649-3p*	0.913	0.001
*miR-2788-3p*	4.47	*miR-7-3p*	0.82	*miR-971-5p*	0.550	*miR-2788-3p*	0.447	*miR-2788-3p*	0.43	*miR-7-3p*	0.857	0.001
*miR-7-3p*	4.56	*miR-2788-3p*	0.84	*miR-2788-3p*	0.642	*miR-3049-5p*	0.479	*miR-193-3p*	0.44	*miR-193-3p*	0.762	0.001
*miR-3049-5p*	4.82	*miR-3049-5p*	0.85	*miR-3049-5p*	0.645	*miR-7-3p*	0.596	*miR-7-3p*	0.68	*miR-971-5p*	0.633	0.003
*miR-216-5p*	7.00	*miR-216-5p*	0.97	*miR-216-5p*	0.719	*miR-216-5p*	0.714	*miR-216-5p*	1.01	*miR-2788-3p*	0.523	0.018
*U6*	8.00	*U6*	1.39	*U6*	1.328	*U6*	0.883	*U6*	1.46	*miR-3049-5p*	0.512	0.021

### Expression stability of candidate reference genes in the thorax + abdomen

The selection of the most stable gene was consistent in all algorithms: all the five methods selected *novel-m0649-3p* as the most stable genes (Table 3). However, the second most stable gene resulted from different algorithms differed slightly from each other. ΔC_t_ method and NormFinder reported *miR-7-3p*, geNorm reported *miR-971-5p* as the second most stable gene, respectively. The comprehensive method, RefFinder, reported *miR-971-5p* as the second most stable gene. Similar to the situation in the head, *U6* also ranked as the least stable genes by all the algorithms except the SD criteria of BestKeeper. According to the SD criteria of BestKeeper, *miR-216-5p* ranked as the least stable gene.

**Table 3:** Ranking of the stability values of the 8 candidate reference genes by the five algorithms in the abodmen + thorax.

RefFinder		ΔC_t_		NormFinder		geNorm		BestKeeper				
Gene	GM	Gene	SV	Gene	SV	Gene	SV	Gene	SD	Gene	[r]	*p* value
*novel-m0649-3p*	1.00	*novel-m0649-3p*	0.95	*novel-m0649-3p*	0.111	*novel-m0649-3p*	0.675	*novel-m0649-3p*	1.16	*novel-m0649-3p*	0.977	0.001
*miR-971-5p*	2.45	*miR-7-3p*	1.04	*miR-7-3p*	0.446	*miR-971-5p*	0.675	*miR-2788-3p*	1.28	*miR-7-3p*	0.972	0.001
*miR-7-3p*	3.13	*miR-971-5p*	1.12	*miR-971-5p*	0.765	*miR-2788-3p*	0.722	*miR-3049-5p*	1.31	*miR-971-5p*	0.916	0.001
*miR-2788-3p*	3.13	*miR-2788-3p*	1.14	*miR-2788-3p*	0.767	*miR-7-3p*	0.791	*miR-971-5p*	1.33	*miR-216-5p*	0.898	0.001
*miR-193-3p*	5.00	*miR-193-3p*	1.18	*miR-193-3p*	0.792	*miR-193-3p*	0.853	*miR-193-3p*	1.33	*miR-2788-3p*	0.892	0.001
*miR-3049-5p*	5.05	*miR-3049-5p*	1.29	*miR-3049-5p*	1.003	*miR-3049-5p*	0.915	*miR-7-3p*	1.50	*miR-193-3p*	0.888	0.001
*miR-216-5p*	7.24	*miR-216-5p*	1.41	*miR-216-5p*	1.118	*miR-216-5p*	1.063	*U6*	1.66	*miR-3049-5p*	0.846	0.001
*U6*	7.74	*U6*	1.75	*U6*	1.597	*U6*	1.236	*miR-216-5p*	1.79	*U6*	0.697	0.001

The number of genes required for normalization in the thorax + abdomen was more than that in the head. Five genes were recommended for normalization, as V_5/6_ was the first number which was less than 0.15 (V_5/6_ = 0.149) (Fig. 2B). However, it is not convenient in practice to use five genes for normalization. The correlation coefficient between *novel-m0649-3p* and *miR-971-5p* was more than 0.9 (*r* = 0.93) (Fig. 3B). Therefore, the combination of *novel-m0649-3p* and *miR-971-5p* for normalization was refused. After the correlation coefficient between *novel-m0649-3p* and other genes was checked, correlation coefficient between *novel-m0649-3p* and *miR-2788-3p* was less than 0.9 (*r* = 0.899). So, combination of *novel-m0649-3p* and *miR-2788-3p* was suggested for normalization.

### Effect of different normalization strategies on *let-7-3p* quantification in the head

**Figure 4:**
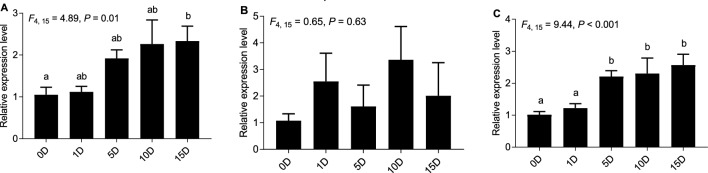
Expression profiles of *let-7-3p* using different reference genes in the head after treating workers with methoprene. (**A**) Expression profile of *let-7-3p* normalized to the most stable reference gene (*novel-m0649-3p*) in the head. (**B**) Expression profile of *let-7-3p* normalized to the least stable reference gene (*U6*) in the head. (**C**) Expression profile of *let-7-3p* normalized to the combination of *novel-m0649-3p* and *miR-193-3p*.

Using different normalization method gave rise to different gene expression results. Significant difference among different assay dates was found using *novel-m0649-3p* as the reference gene (*F*_4, 15_ = 4.89, *P* = 0.01), while no significant difference was found using *U6* as the reference gene (*F*_4, 15_ = 0.65, *P* = 0.63) in the head (Fig. 4A, B). Smaller *P* value was obtained using the combination of *novel-m0649-3p* and *miR-193-3p* for normalization than that using *novel-m0649-3p* only in the head (Fig. 4C). According to Fig. 4, *let-7-3p* exhibited a clear rising pattern when normalized to the most stable reference gene, or the combination of the two most stable reference genes in comparison with when normalized to the least stable reference gene *U6*. No clear expression pattern of *let-7-3p* was observed using the *U6* as the normalization gene.

### Effect of different normalization strategies on *let-7-3p* quantification in the thorax + abdomen

**Figure 5:**
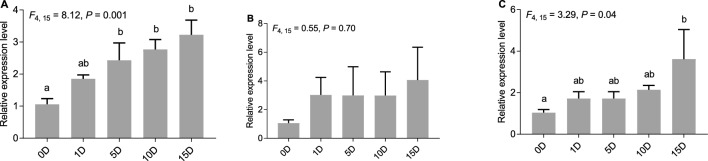
Expression profiles of *let-7-3p* using different reference genes in the thorax + abdomen after treating workers with methoprene. (**A**) Expression profile of *let-7-3p* normalized to the most stable reference gene (*novel-m0649-3p*) in the thorax + abdomen. (**B**) Expression profile of *let-7-3p* normalized to the least stable reference gene (*U6*) in the thorax + abdomen. (**C**) Expression profile of *let-7-3p* normalized to the combination of *novel-m0649-3p* and *miR-2788-3p*.

There were similar results in the thorax + abdomen using *novel-m0649-3p* as the reference gene: significant difference was found (*F*_4, 15_ = 8.12, *P* = 0.001), while no significant difference was found using *U6* as the reference gene (*F*_4, 15_ = 0.55, *P* = 0.70) (Fig. 5A, B). However, larger *P* value was observed for the combination of *novel-m0649-3p* and *miR-2788-3p* than that for *novel-m0649-3p* only in the thorax + abdomen (Fig. 5C).

## Discussion

RT-qPCR is a powerful and sensitive tool for gene quantification. Normalization to a reference gene which is stably expressed is a popular method to eliminate the error in the relative quantification method. However, there are no genes whose expression is stable in all the experimental conditions. As more attention was paid to the influence of reference gene on the quantification results, a range of reference genes screening researches were conducted both for the mRNA and miRNA quantification. Here, we screened the reference genes for miRNA expression analysis in the study of soldier caste differentiation. Our data showed that *novel-m0649-3p* was the most stable reference gene both in the head and thorax + abdomen. *U6*, which is widely used for normalization of miRNA quantification data, was not a good reference gene. In addition, normalization to two reference genes was better than that to one reference gene in the head. More variation of the Ct values was observed in the thorax + abdomen than that in the head. The most obvious changes after treatment of workers with methoprene happen in the abdomen, such as enlargement of fat body^44^, massive expression of the hexamerin^8^ etc. Therefore, more response genes are expected to be observed in the thorax + abdomen. Colony response variability was observed in *C. formosanus* after methoprene treatment^35^, implying gene expression variability at the colony level. Therefore, this may be one reason for more variation in the thorax + abdomen.

*Let-7* is first discovered in *Caenorhabditis elegans* (Maupas) as a gene switcher. Its function is conserved across species^45^. The expression levels of *let-7-3p* was analyzed using different reference genes in our study. *U6* is used widely as reference gene. However, our analysis recognizes *U6* as the least stable reference gene. When our data were normalized to *U6*, the data became more scattered in comparison to normalizing to *novel-m0649-3p* gene, and no significant difference was found. In contrast, using *novel-m0649-3p* as the reference gene produced a clear rising pattern of *let-7-3p*. The pattern of expression profile of *let-7-3p* was different using the most stable and the least stable reference genes to normalize the data, which demonstrates the importance of choosing the suitable reference genes for normalization of the qPCR data. So, it is suggested to screen the reference gene for the specific purpose.

Although all the algorithms identified *novel-m0649-3p* as the most stable reference genes, the ranking of other genes was slightly different by different algorithms. Different algorisms returned slightly different results, as the underlying principles vary among the five algorithms. geNorm calculates the gene stability value M to represent the stability of the expression of the gene. The less the value of M, the more stable of the gene. The pairwise variation value (V value) is introduced to determine the number of the reference genes needed. If V_n_/V_n+1_ is less than 0.15, number of n genes is suggested for normalization. In the head, V_2/3_ was less than 0.15, so a combination of 2 genes was enough for normalization. However, combination of 5 genes was needed for normalization in the thorax + abdomen, as V_5/6_ = 0.149. Nevertheless, it is not convenient to use 5 genes for normalization. The combination of two genes was still recommended for normalization in the thorax + abdomen. As suggested by Babion et al*.*^19^, the combination of two genes for normalization should not be highly correlated. So, in the thorax + abdomen, combination of *novel-m0649-3p* and *miR-2788-3p* was suggested for normalization. The combination of two genes for normalization gave rise to smaller *P* value compared to normalization to one gene in the head. However, because the two reference genes in the combination were closely correlated with each other in the abdomen+thorax, using two reference genes did not lead to better results than using the most stable reference gene. Normfinder estimates the stability value of a candidate reference genes by calculating the intragroup and intergroup variations of the candidate reference genes. The stability value represents the systematic error using a certain gene for normalization. However, Normfinder requires larger sample size than geNorm. Bestkeeper calculates two parameters as the gene stability criteria. One is the standard deviation of the C_t_ value; another is the correlation coefficient between the candidate reference gene and the BestKeeper Index. Although difference in the gene stability ranking was observed for the five algorithms, the results obtained by different algorisms were somewhat similar, which indicates that the five algorithms have the capability to differentiate the relative stability of the candidate reference genes.

There are two kinds of methods for qPCR: the dye-based method and the probe-based method. The probe-based method is more sensitive and more specific compared with the dye-based method. So, the probe-based method was adopted in our project. In order to acquire the template for qPCR, reverse transcription of miRNAs is needed. There are two kinds of primers for reverse transcription of miRNAs: stem-loop RT primer and poly(A)-tailing RT primer. According to our preliminary results, poly(A)-tailing method was likely to produce negative results, because there was unspecific amplification according to the melting curve and the gel image of the agarose gel electrophoresis of the qPCR products. Therefore, the stem-loop RT primer was used for the reverse transcription of miRNAs in our project. Chen et al*.*^36^ suggested using 50 nM stem-loop RT primers in the probe-based method, while Kramer^46^ suggested using 1 nM of the primer. According to our preliminary experiment, the concentration of 30 nM stem-loop RT primers led to more sensitive and more efficient amplification during qPCR. As the RT primer has the binding site of the reverse primer of qPCR, too high a concentration of the RT primer may lead to the binding of the reverse primer with the RT primer, which results in less efficient amplification.

Our study has established normalization strategies for expression analysis of miRNAs in the study of solider caste differentiation in *C. formosanus*. It is suggested to use *novel-m0649-3p* or the combination of *novel-m0649-3p* and *miR-193-3p* for normalization in the head, and to use *novel-m0649-3p* as the reference gene in the thorax + abdomen. The study of the differentiation mechanism is mainly focused at the mRNA and proteins level. miRNAs are important post-transcriptional regulators. There is still a gap in the studies of the roles of miRNAs in soldier caste regulation. Screening of the suitable reference genes establishes the first step to study the miRNA expression level during solider caste differentiation. Further studies of the roles of miRNAs in soldier caste differentiation will widen our understanding of the caste differentiation in termites.

## Supplementary Information

Supplementary Information.

## Data Availability

The miRNome data were submitted to GenBank Sequence Read Archive databases under the BioProject PRJNA922245.
