# Supplementary material for: Screening of reference genes for microRNA analysis in the study of solider caste differentiation of Formosan subterranean termite Coptotermes formosanus Shiraki
Source: Sci Rep. 2023 Jun 9;13:9399. doi: 10.1038/s41598-023-35926-7 (PMC10256727; doi:10.1038/s41598-023-35926-7)
Supplement: Supplementary file 1 — Supplementary Information. [file 41598_2023_35926_MOESM1_ESM.docx]

|  | *miR-193-3p* | *miR-971-5p* | *miR-3049-5p* | *miR-216-5p* | *miR-7-3p* | *novel-m0649-3p* | *miR-2788-3p* | *U6* |
| --- | --- | --- | --- | --- | --- | --- | --- | --- |
| Bioassay date | 0.96 | 0.98 | 0.96 | 0.85 | 0.94 | 0.77 | 0.85 | 0.68 |
| Body part | < 0.001 | < 0.001 | < 0.001 | < 0.001 | < 0.001 | 0.007 | < 0.001 | 0.96 |
| Interaction effect | 0.77 | 0.98 | 0.88 | 0.52 | 0.91 | 0.79 | 0.90 | 0.61 |

Table S1. Effect of bioassay date and body part on C_t_ values of the 8 candidate reference genes.
